# Supplementary material for: Potential impact of outpatient stewardship interventions on antibiotic exposures of common bacterial pathogens
Source: eLife. 2020 Feb 5;9:e52307. doi: 10.7554/eLife.52307 (PMC7025820; doi:10.7554/eLife.52307)
Supplement: Figure 1—source data 1. [file elife-52307-fig1-data1.docx]

**Figure 1 – Source Data File 1.** Summary of etiology studies.

| **Publication** | **Study description** | **Etiology results** |
| --- | --- | --- |
| Bacteriology of Acute Otitis Media in Adults (Celin et al., 1991) | 34 outpatients aged 18 years or older presenting at the emergency department and diagnosed with acute otitis media; middle-ear aspirates were collected for culture | *H. influenzae*: 26%  *M. catarrhalis*: 3%  *S. aureus*: 3%  *S. pneumoniae*: 21%  *S. pyogenes*: 3% |
| Ten-year review of otitis media pathogens (Bluestone et al., 1992) | 2807 effusions from children with acute otitis media were collected | *H. influenzae*: 23%  *M. catarrhalis*: 14%  *S. aureus*: 1%  *P. aeruginosa*: 1%  *S. pneumoniae*: 35%  *S. pyogenes*: 3% |
| The microbial etiology and antimicrobial therapy of adults with acute community-acquired sinusitis: A fifteen-year experience at the University of Virginia and review of other selected studies (Gwaltney et al., 1992) | 339 adult patients with acute community-acquired sinusitis; sinus aspirates were collected prior to treatment | To estimate the proportion of acute sinusitis cases caused by each organism, we multiplied the proportion of bacterial cultures in which each organism was identified (shown below) by the proportion of all acute sinusitis cases estimated to be bacterial in nature (2%) (Fokkens et al., 2007):  *H. influenzae*: 35%  *M. catarrhalis*: 4%  *S. aureus*: 3%  *S. pneumoniae*: 41% |
| Microbiology and Management of Chronic Maxillary Sinusitis (Brook et al., 1994) | 68 patients aged 18 to 67 years who underwent the Caldwell-Luc procedure for chronic sinusitis and whose cultures showed bacterial growth; specimens were taken from the infected sinus during surgery | *E. coli*: 2.9%  *H. influenzae*: 4.4%  *K. pneumoniae*: 2.9%  *M. catarrhalis*: 11.8%  *S. aureus*: 11.8%  *S. agalactiae*: 5.9%  *S. pneumoniae*: 5.9%  *S. pyogenes*: 7.4% |
| Increasing Prevalence of Antimicrobial Resistance Among Uropathogens Causing Acute Uncomplicated Cystitis in Women (Gupta et al., 1999) | 4802 women aged 18 to 50 years with an outpatient diagnosis of acute cystitis; urine samples were collected | Female  *E. coli*: 86%  *Klebsiella* spp.: 3% |
| Etiology and treatment of community-acquired pneumonia in ambulatory children (Wubbel et al., 1999) | 174 ambulatory patients age 6 months to 16 years with radiographic and clinical evidence of pneumonia; blood, nasopharyngeal, and pharyngeal samples were collected | *S. pneumoniae*: 27% |
| *Staphylococcus aureus* is the most common identified cause of cellulitis: a systematic review (Chira & Miller, 2010) | 808 patients with cellulitis who were included in 16 studies published between 1966 and 2006; studies were eligible for inclusion if they used needle aspiration and/or punch biopsy and excluded if they pertained to ocular, odontogenic, pelvic, or surgical site-associated cellulitis | *E. coli*: 0.4%  *H. influenzae*: 0.1%  *P. aeruginosa*: 0.5%  *S. aureus*: 8%  *S. agalactiae*: 0.5%  *S. pyogenes*: 4.3% |
| Antibiotic Resistance Patterns of Outpatient Pediatric Urinary Tract Infections (Edlin et al., 2013) | 25,418 urinary isolates from patients younger than 18 years in the outpatient setting from 195 United States hospitals in The Surveillance Network | Female  *E. coli*: 83%  *Klebsiella* spp.: 4%  *P. aeruginosa*: 2%  Male  *E. coli*: 50%  *Klebsiella* spp.: 10%  *P. aeruginosa*: 7% |
| Community-Acquired Pneumonia Requiring Hospitalization among U.S. Adults (Jain et al., 2015) | 2259 patients who were hospitalized for community-acquired pneumonia (based on radiographic evidence) and had specimens for both bacterial and viral testing; specimens may have been taken from blood, acute-phase serum, urine, nasopharynx, oropharynx, sputum, pleural fluid, endotracheal aspirate, or broncho-alveolar-lavage samples | *H. influenzae*: 0.6%  *Pseudomonas* spp.: 0.4%  *S. aureus*: 1.6%  *S. pneumoniae*: 5.1%  *S. pyogenes*: 0.3% |

**References**

Bluestone, C. D., Stephenson, J. S., & Martin, L. M. (1992). Ten-year review of otitis media pathogens. *The Pediatric Infectious Disease Journal*, *11*(8), S7–S11. https://doi.org/10.1097/00006454-199208001-00002

Brook, I., Thompson, D. H., & Frazier, E. H. (1994). Microbiology and Management of Chronic Maxillary Sinusitis. *Archives of Otolaryngology–Head & Neck Surgery*, *120*(12), 1317–1320.

Celin, S. E., Bluestone, C. D., Stephenson, J., Yilmaz, H. M., & Collins, J. J. (1991). Bacteriology of Acute Otitis Media in Adults. *JAMA*, *266*(16), 2249–2252. https://doi.org/10.1001/jama.266.16.2249

Chira, S., & Miller, L. G. (2010). *Staphylococcus aureus* is the most common identified cause of cellulitis: A systematic review. *Epidemiology and Infection*, *138*(3), 313–317. https://doi.org/10.1017/S0950268809990483

Edlin, R. S., Shapiro, D. J., Hersh, A. L., & Copp, H. L. (2013). Antibiotic resistance patterns of outpatient pediatric urinary tract infections. *The Journal of Urology*, *190*(1), 222–227. https://doi.org/10.1016/j.juro.2013.01.069

Fokkens, W., Lund, V., & Mullol, J. (2007). European position paper on rhinosinusitis and nasal polyps 2007. A summary for otorhinolaryngologists. *Rhinology*, *45*(97).

Gupta, K., Scholes, D., & Stamm, W. E. (1999). Increasing prevalence of antimicrobial resistance among uropathogens causing acute uncomplicated cystitis in women. *JAMA*, *281*(8), 736–738. https://doi.org/10.1001/jama.281.8.736

Gwaltney, J. M., Scheld, W. M., Sande, M. A., & Sydnor, A. (1992). The Microbial Etiology and Antimicrobial Therapy of Adults with Acute Community-Acquired Sinusitis—A 15-Year Experience at the University of Virginia and Review of Other Selected Studies. *Journal of Allergy and Clinical Immunology*, *90*(3), 457–462. https://doi.org/10.1016/0091-6749(92)90169-3

Jain, S., Self, W. H., Wunderink, R. G., Fakhran, S., Balk, R., Bramley, A. M., Reed, C., Grijalva, C. G., Anderson, E. J., Courtney, D. M., Chappell, J. D., Qi, C., Hart, E. M., Carroll, F., Trabue, C., Donnelly, H. K., Williams, D. J., Zhu, Y., Arnold, S. R., … Finelli, L. (2015). Community-Acquired Pneumonia Requiring Hospitalization among U.S. Adults. *New England Journal of Medicine*, *373*(5), 415–427. https://doi.org/10.1056/NEJMoa1500245

Wubbel, L., Muniz, L., Ahmed, A., Trujillo, M., Carubelli, C., McCoig, C., Abramo, T., Leinonen, M., & McCracken, G. H. (1999). Etiology and treatment of community-acquired pneumonia in ambulatory children. *The Pediatric Infectious Disease Journal*, *18*(2), 98–104.
