# Supplementary material for: Potential impact of outpatient stewardship interventions on antibiotic exposures of common bacterial pathogens
Source: eLife. 2020 Feb 5;9:e52307. doi: 10.7554/eLife.52307 (PMC7025820; doi:10.7554/eLife.52307)
Supplement: Figure 1—source data 2. — Modifications were made if the reported proportion of bacterial cases for a given condition exceeded the estimated proportion of appropriate prescriptions reported in Fleming-Dutra et al. (2016). [file elife-52307-fig1-data2.docx]

**Figure 1 – Source Data File 2.** Modifications to proportions of unnecessary antibiotic prescriptions based on published etiology studies and antibiotic use in NAMCS/NHAMCS. Modifications were made if the estimated proportion of bacterial cases for a given condition exceeded the estimated proportion of appropriate prescriptions reported in Fleming-Dutra et al. (Fleming-Dutra et al., 2016).

| **Age group** | **Fleming-Dutra estimate** | **New estimate** | **Explanation** |
| --- | --- | --- | --- |
| ***Pharyngitis*** | | | |
| 0-19 years old | 34% | Pharyngitis: 47%;  Strep throat: 0% | Assuming that all strep-associated use of our antibiotics of interest (based on NAMCS/NHAMCS) was necessary, we estimated the proportion of non-strep pharyngitis antibiotic use that would have to be eliminated to maintain the overall estimate reported by Fleming-Dutra et al. (Fleming-Dutra et al., 2016). |
| 20-64 years old | 75% | Updated to 51% overall; Pharyngitis: 100%;  Strep throat: 0% |  |
| >64 years old | NA (assume 0%) | NA (assume 0%) | No change. |
| ***Sinusitis*** | | | |
| 0-19 years old | 9% | Acute: 18%;  Chronic: 0% | While acute sinusitis is rarely bacterial, bacteria are believed to play a much more prominent role in the etiology of chronic sinusitis (Brook, 2016). Thus, we assume that antibiotic use would only be eliminated from acute sinusitis and maintain the overall estimate of unnecessary use reported by Fleming-Dutra et al.(Fleming-Dutra et al., 2016) when possible. |
| 20-64 years old | 51% | Updated to 40% overall;  Acute: 100%;  Chronic: 0% |  |
| >64 years old | 16% | Acute: 34%;  Chronic: 0% |  |
| ***Suppurative otitis media*** | | | |
| 0-19 years old | 10% | 10% | No change. Approximately 84% of pediatric cases were found to have bacterial growth (Bluestone, Stephenson, & Martin, 1992), so 10% is a reasonable estimate for avertable use. |
| 20-64 years old | 33% | 26% | Based on an etiology study in adults, only 26% of cases were found to have no bacterial growth (Celin, Bluestone, Stephenson, Yilmaz, & Collins, 1991). |
| >64 years old | NA (assume 0%) | NA (assume 0%) | No change. |

**References**

Bluestone, C. D., Stephenson, J. S., & Martin, L. M. (1992). Ten-year review of otitis media pathogens. *The Pediatric Infectious Disease Journal*, *11*(8), S7–S11. https://doi.org/10.1097/00006454-199208001-00002

Brook, I. (2016). Microbiology of chronic rhinosinusitis. *European Journal of Clinical Microbiology & Infectious Diseases*, *35*(7), 1059–1068. https://doi.org/10.1007/s10096-016-2640-x

Celin, S. E., Bluestone, C. D., Stephenson, J., Yilmaz, H. M., & Collins, J. J. (1991). Bacteriology of Acute Otitis Media in Adults. *JAMA*, *266*(16), 2249–2252. https://doi.org/10.1001/jama.266.16.2249

Fleming-Dutra, K. E., Hersh, A. L., Shapiro, D. J., Bartoces, M., Enns, E. A., File, T. M., … Hicks, L. A. (2016). Prevalence of Inappropriate Antibiotic Prescriptions Among US Ambulatory Care Visits, 2010-2011. *JAMA*, *315*(17), 1864–1873. https://doi.org/10.1001/jama.2016.4151
