# Supplementary material for: Potential impact of outpatient stewardship interventions on antibiotic exposures of common bacterial pathogens
Source: eLife. 2020 Feb 5;9:e52307. doi: 10.7554/eLife.52307 (PMC7025820; doi:10.7554/eLife.52307)
Supplement: Figure 1—source data 3. [file elife-52307-fig1-data3.docx]

**Figure 1 – Source Data File 3.** Estimated antibiotic class exposures by exposed and causative species.

The following tables contain the estimated number of annual antibiotic exposures (in 100,000s) in the outpatient setting in the United States by the species being exposed (y-axis) and the species that is the cause of antibiotic use (x-axis). The values in each table, excluding the “Other” and “Overall” columns were calculated using the following expression:

$$\sum_{g=1}^{G} \sum_{i=0}^{I} d_{aig}\times e_{s_{c}ig}\times p_{s_{e}0g}$$

Let *a* represent antibiotic class, *s_c_* represent causative species, *s_e_* represent exposed species, *i* represent ICD-9-CM diagnosis code, and *g* represent age group (under 1 year, 1-5 years, 6-19 years, 20-64 years, 65 years old and over). Then, $d_{aig}$ is the number of prescriptions of antibiotic class *a* associated with diagnosis code *i* in age group *g*, $e_{s_{c}ig}$ is the proportion of cases with diagnosis code *i* that are estimated to be caused by causative species *s_c_* in age group *g*, and $p_{s_{e}0g}$ is the healthy carriage prevalence of exposed species *s_e_* among individuals in age group *g*. When *s_c_* and *s_e_* are the same organism, $p_{s_{e}0g}$ is set to 1. For each variable, we use the same data sources and estimation methods described in the main text (NAMCS/NHAMCS 2015, Human Microbiome Project, and published literature).

The diagonals, shaded in light gray, are “target” antibiotic exposures for the pathogen of interest; in other words, these are antibiotic exposures that the organism experienced because it was causing disease. The “Overall” column is all exposures to the antibiotic class of interest experienced by each species and is equivalent to *T_as_* in Equation 1 of the main text. The “Other” column is the difference between the “Overall” column and the sum of the remaining columns. Note that this column represents exposures due to non-bacterial causes as well as bacterial organisms other than our nine potential pathogens of interest.

The “Any antibiotic” table includes all prescriptions for drugs classified as antibiotics based on the Lexicon Plus® Therapeutic Classification Scheme (*Lexicon Plus*, 2008). Any drug with a Level 1 description of “anti-infectives”, but with a Level 2 description that was not “amebicides”, “antihelmintics”, “antifungals”, “antimalarial agents”, or “antiviral agents” was included as an antibiotic. The “Any included antibiotic class” table includes all prescriptions for drugs in the classes shown in the following tables. Antibiotic classes were defined using the Level 2 categories in Lexicon Plus®.

These tables are designed to facilitate a better understanding of the “nuts and bolts” of this work and to enable calculation of other metrics of interest. For example, the proportion of bystander exposures as calculated by the authors in an earlier paper (Tedijanto, Olesen, Grad, & Lipsitch, 2018) for any given antibiotic and species pair can be calculated as one minus the target exposures (diagonal value) over the “Overall” value. These tables can also be used to understand how interventions focused on a single species (e.g. vaccines) may reduce antibiotic exposures experienced by another species.

**
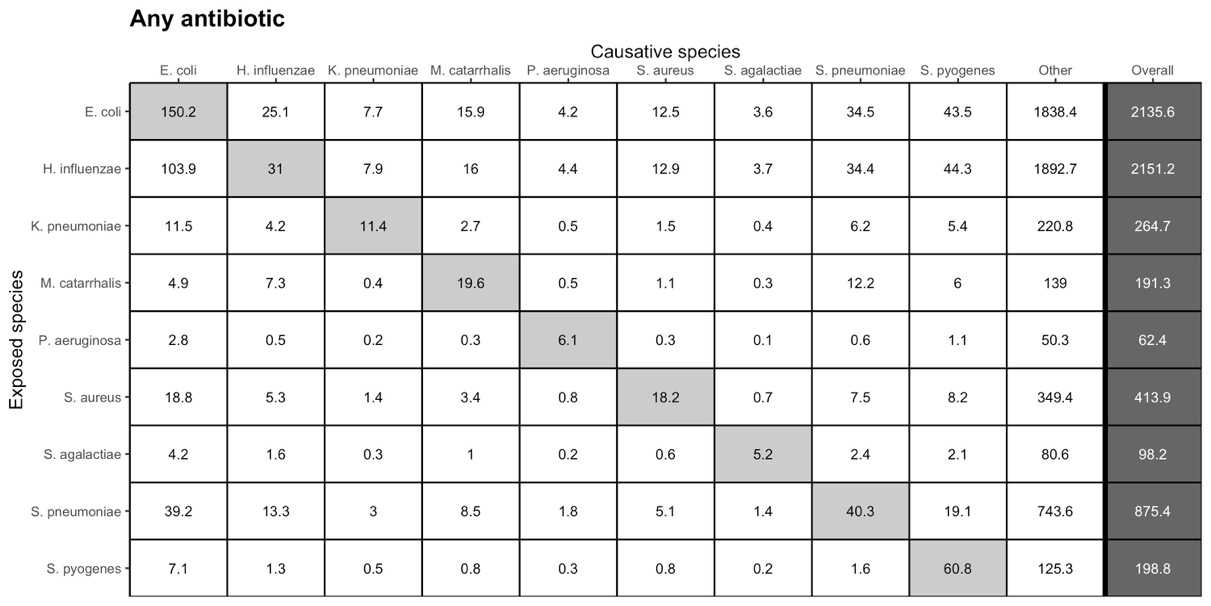
**

**
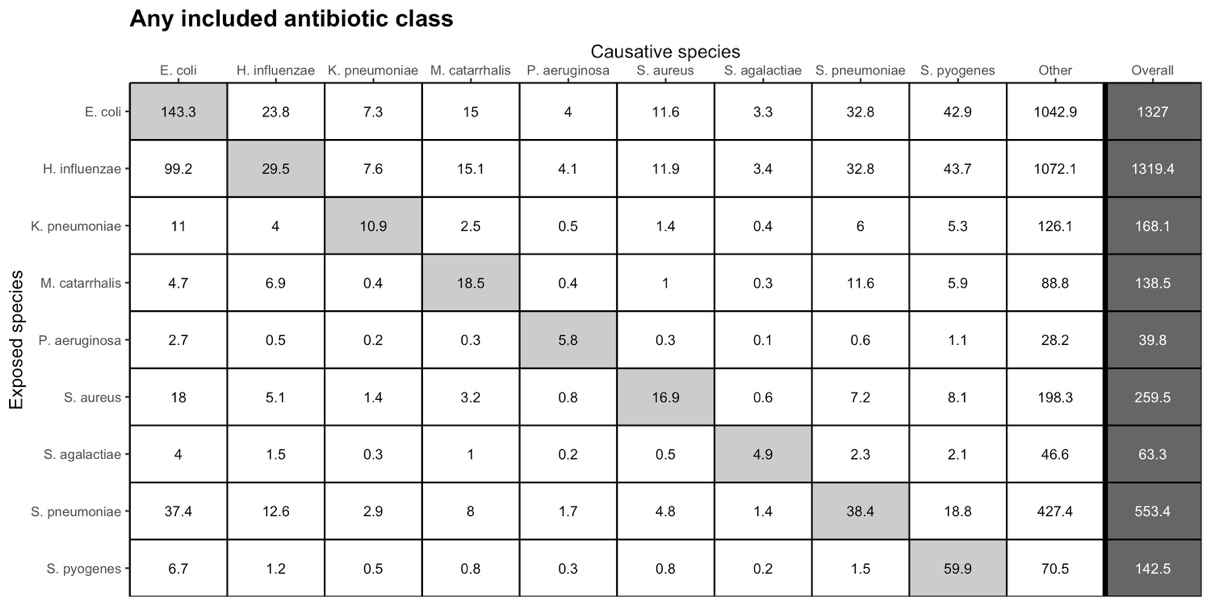
**

**
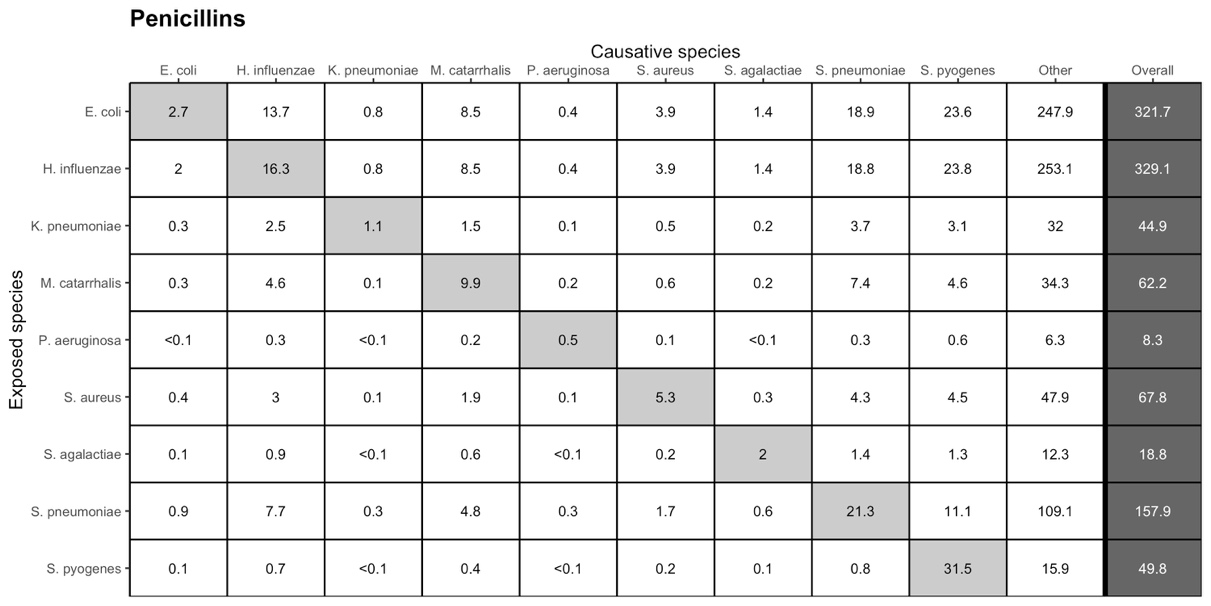
**

**
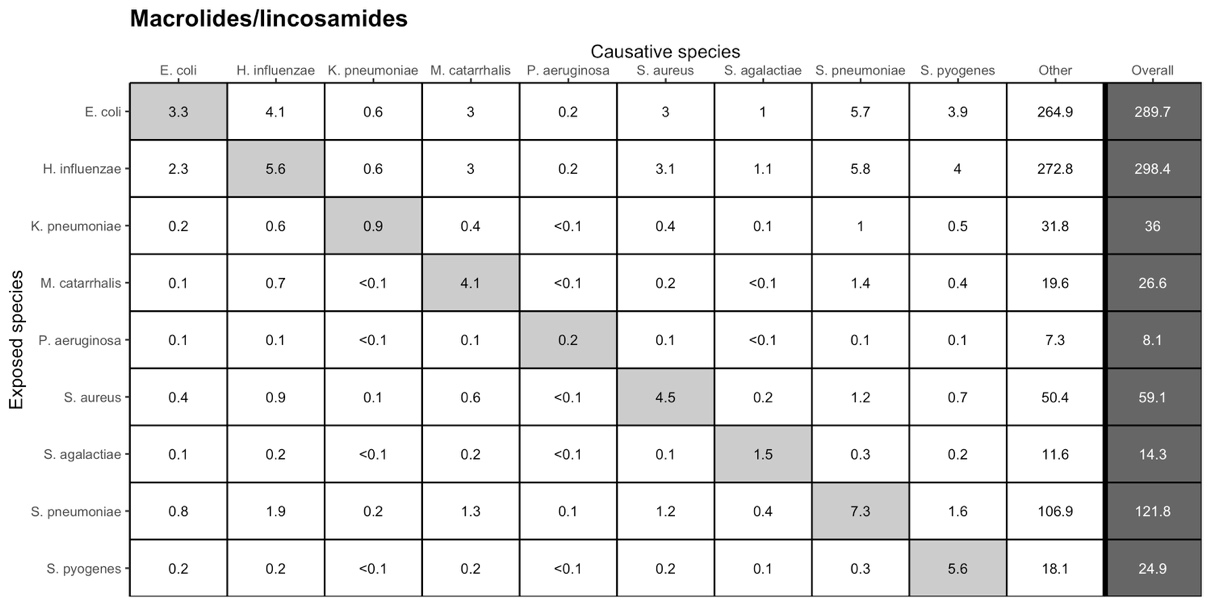
**

**
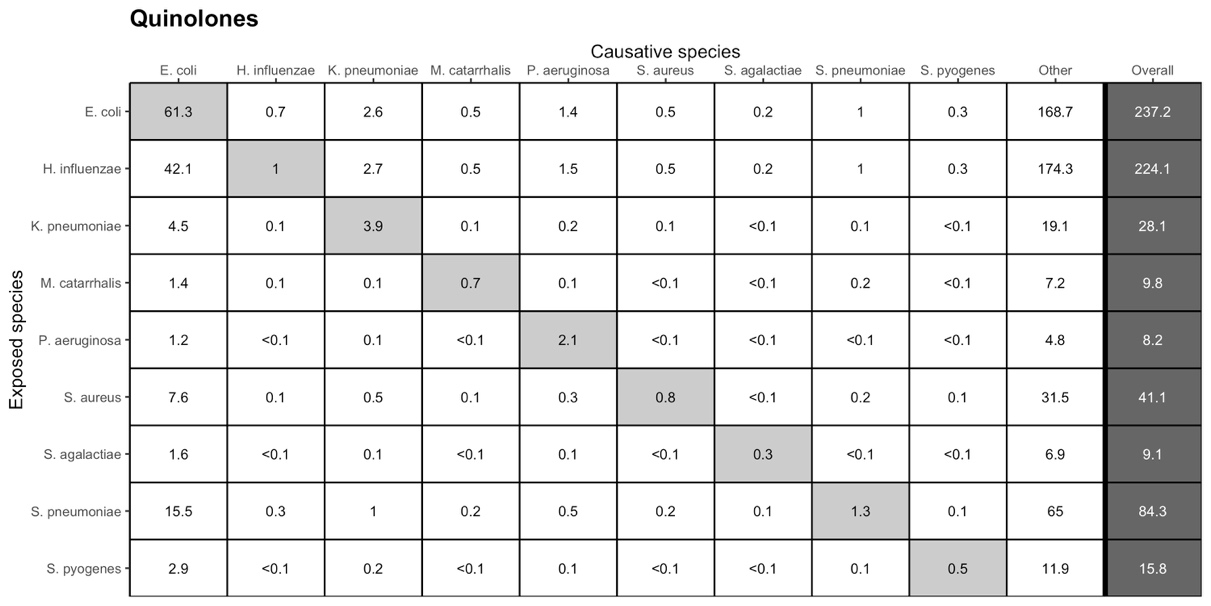
**

**
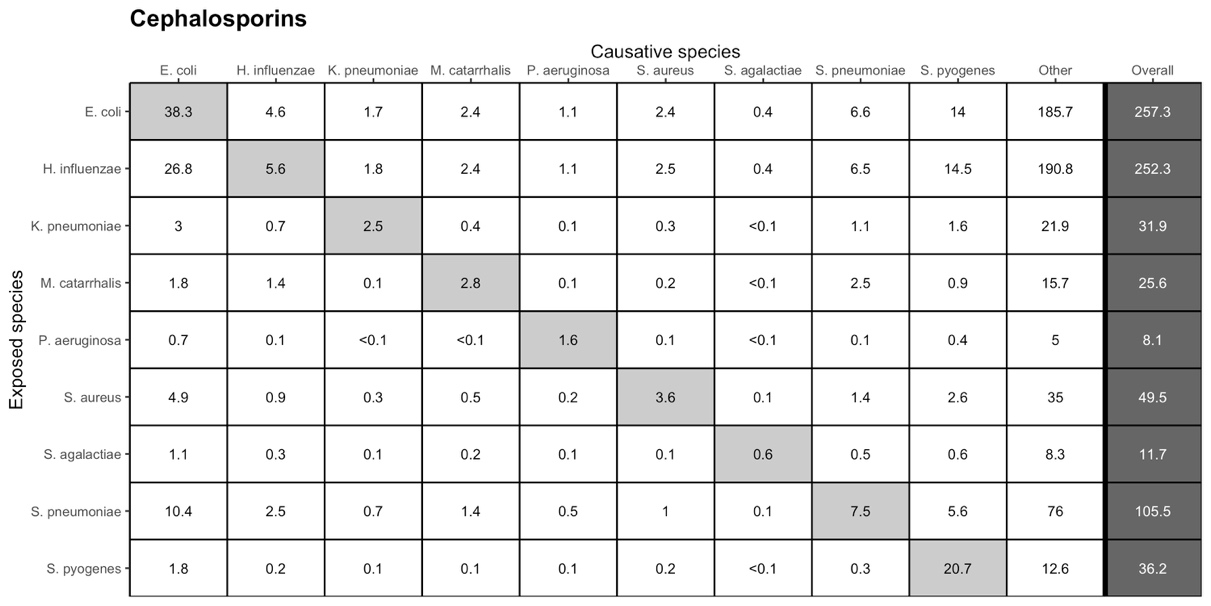
**

**
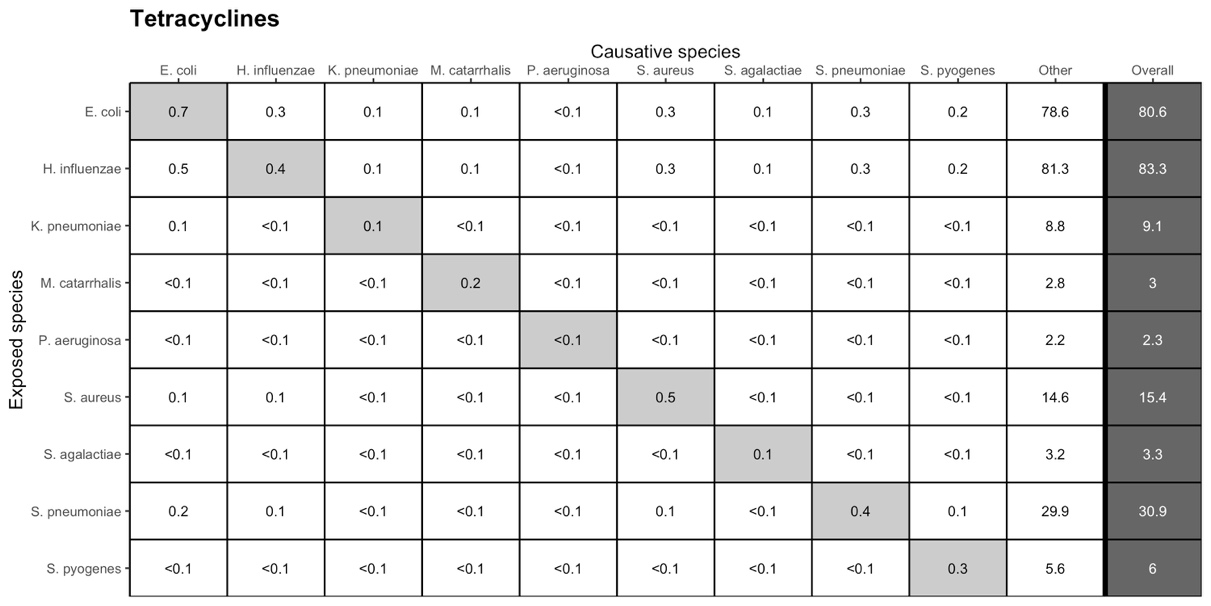
**

**
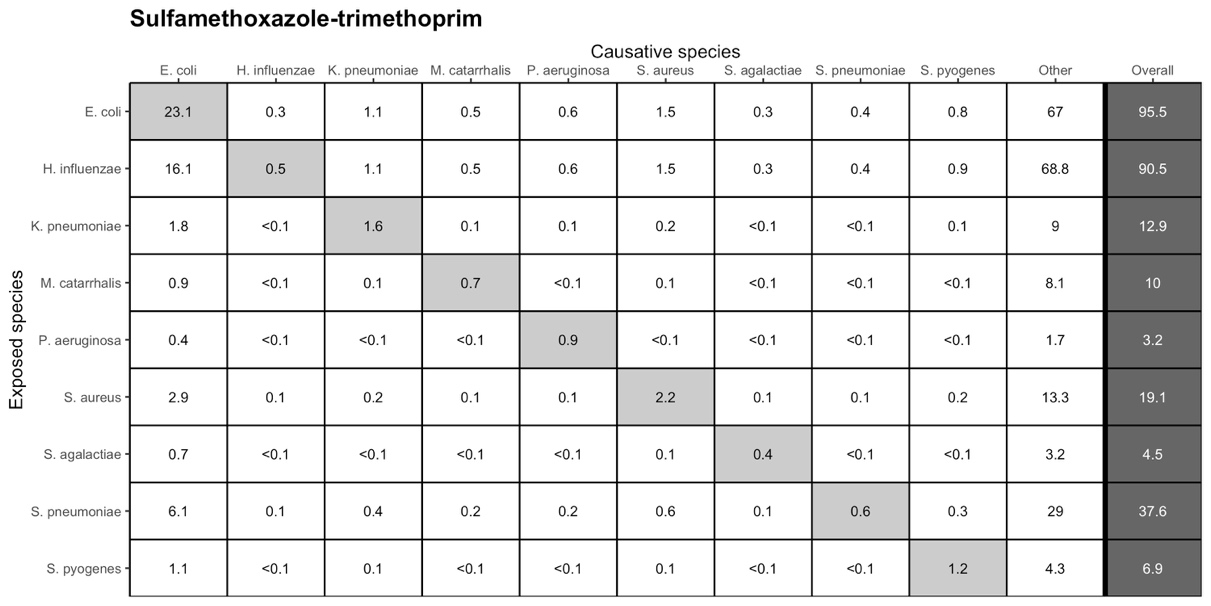
**

**
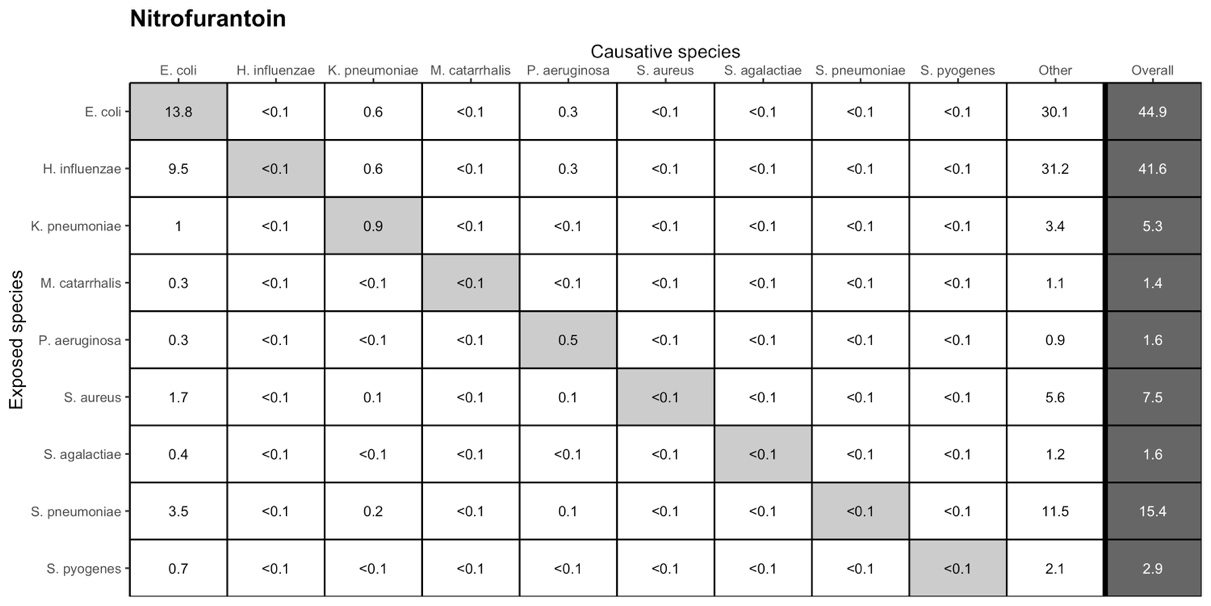
**

**References**

*Lexicon Plus*. (2008). Retrieved from https://www.cerner.com/solutions/drug-database

Tedijanto, C., Olesen, S. W., Grad, Y. H., & Lipsitch, M. (2018). Estimating the proportion of bystander selection for antibiotic resistance among potentially pathogenic bacterial flora. *Proceedings of the National Academy of Sciences*, *115*(51), E11988–E11995. https://doi.org/10.1073/pnas.1810840115
